# Sex estimation from morphometric measurements of os coxae in computed tomography images and validity and accuracy of “diagnose sexuelle probabiliste V2” software for the Turkish population

**DOI:** 10.1007/s00414-026-03801-5

**Published:** 2026-04-15

**Authors:** İlknur Çöllü, Sinan Bakırcı

**Affiliations:** İzmir Kâtip Çelebi University, Faculty of Medicine, Department of Anatomy, İzmir, Türkiye

**Keywords:** Diagnose sexuelle probabiliste, DSP2, Probabilistic sex diagnosis, hip bone, Sexing rate, Sex estimation, Accuracy rate

## Abstract

**Objective:**

This study aimed to analyze the reliability, applicability, and accuracy of the “Diagnose Sexuelle Probabiliste V2” software used for sex estimation on pelvic CT images of the Turkish population.

**Methods:**

Pelvic CT images of 200 individuals (100 male, 100 female) were used. Slicer 5.8.0 software was used to create a three-dimensional representation of the bone and perform the measurements. 10 morphometric measurements were made on the os coxae to estimate the sex of the individuals on the images.

**Results:**

Of the 10 variables used in DSP2, the difference between the sexes was significant for all variables except SA (*p* < 0.001). When the posterior probability threshold was set at 0.95 or higher, the sex of only 3 individuals could not be estimated using the 10 variables in DSP2. The sexing success rate was 98.5%. The accuracy rate was 100%.

**Conclusion:**

DSP2 is a highly reliable method for sex estimation from pelvic CT images for the Turkish population.

## Introduction

Sex estimation plays a crucial role in the identification process in forensic medicine and anthropology. The os coxae, due to its pronounced sexual dimorphism, is one of the bones frequently used for sex estimation. It also holds a significant place in anthropological and forensic studies [1, 2]. DSP2 (Diagnose Sexuelle Probabiliste v2), one of the software programs developed for this purpose, has been tested on dry bones and radiological images in different populations and has been reported as a method with high accuracy and validity [3–8]. Studies using DSP2 have been applied to diverse populations such as Spain [4], Romania [5], Greece [6], Belgium [7], Denmark [9], and France [8], and reliable results have been obtained. Furthermore, it is known that genetic variation, environmental factors, and lifestyle differences among populations influence bone morphology. Due to this discrepancy, our study aimed to analyze the reliability, applicability, and accuracy of the DSP2 method in the Turkish population and to compare sexing and accuracy rates reported in different populations.

## Materials and methods

**Fig. 1:**
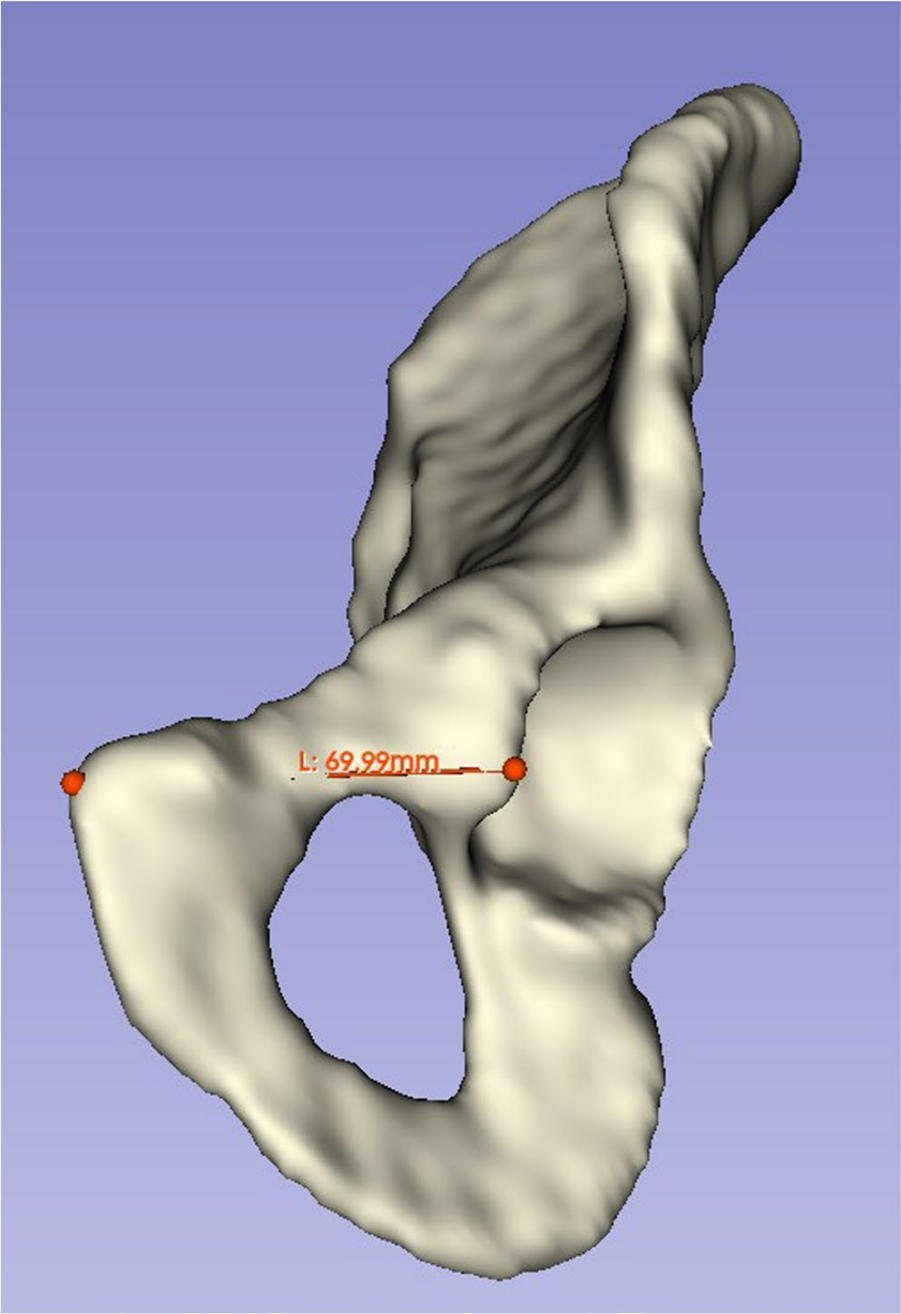
PUM: Acetabulo-symphyseal pubic length

**Fig. 2:**
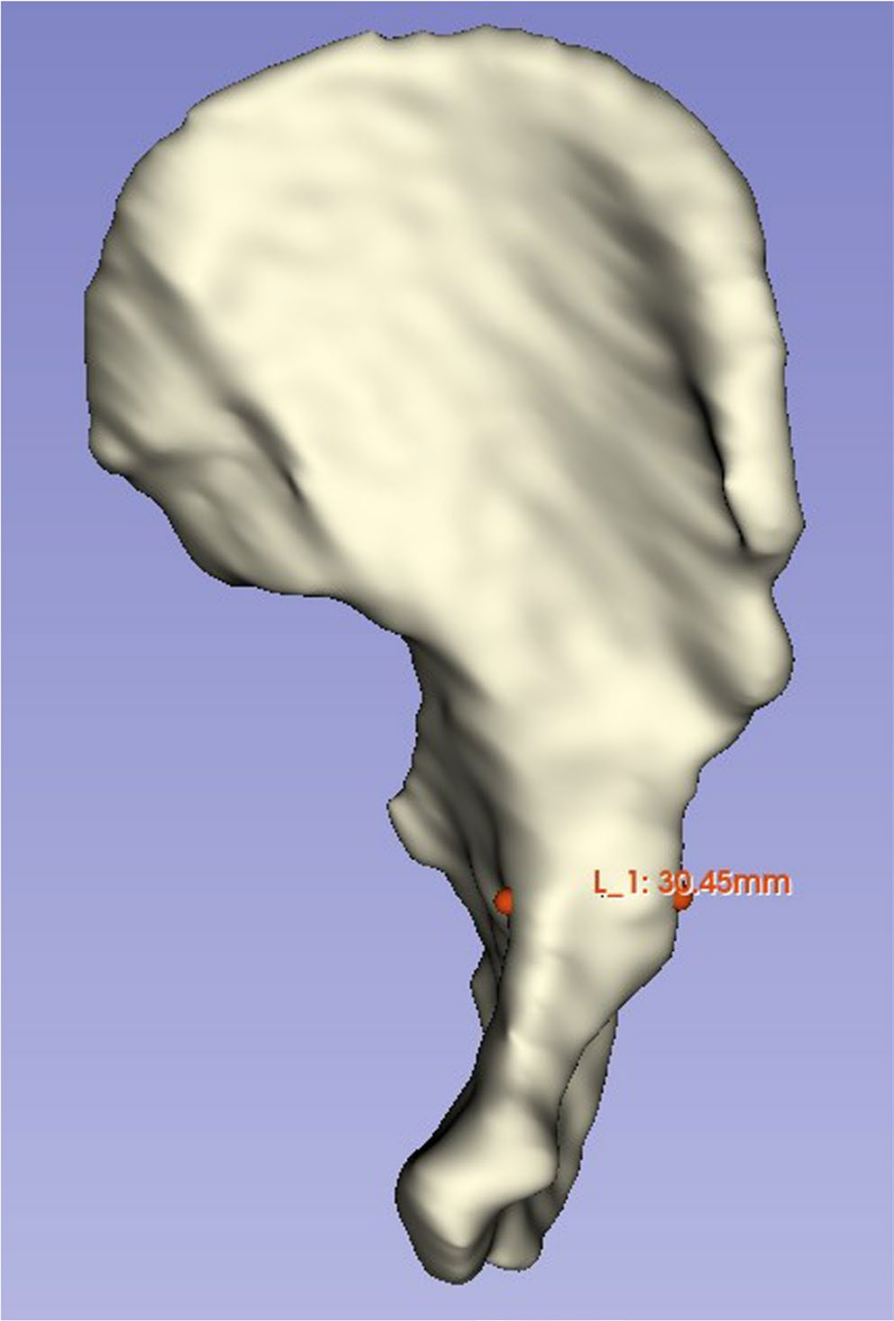
SPU: Cotylo-pubic breadth

**Fig. 3:**
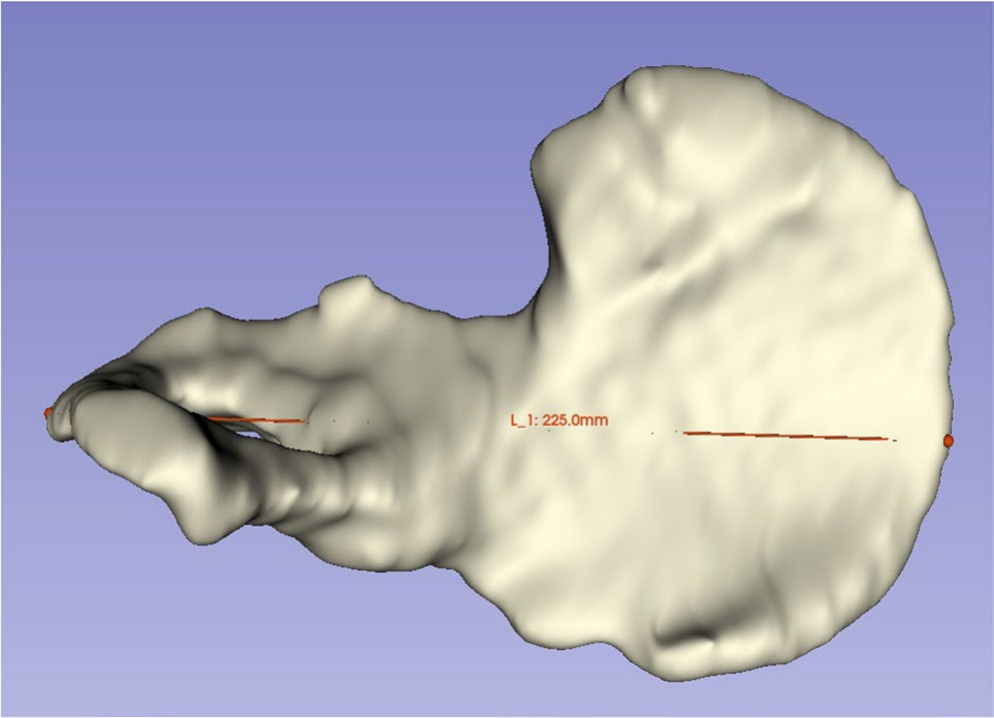
DCOX: Maximum pelvic height

**Fig. 4:**
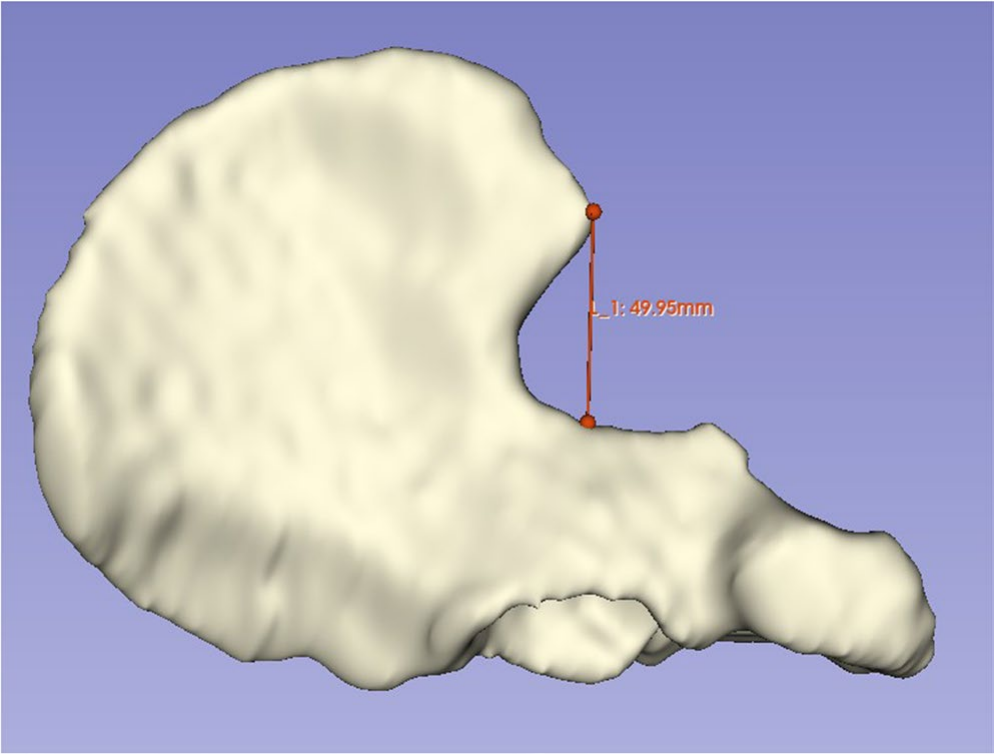
IIMT: Height of the greater sciatic notch

**Fig. 5:**
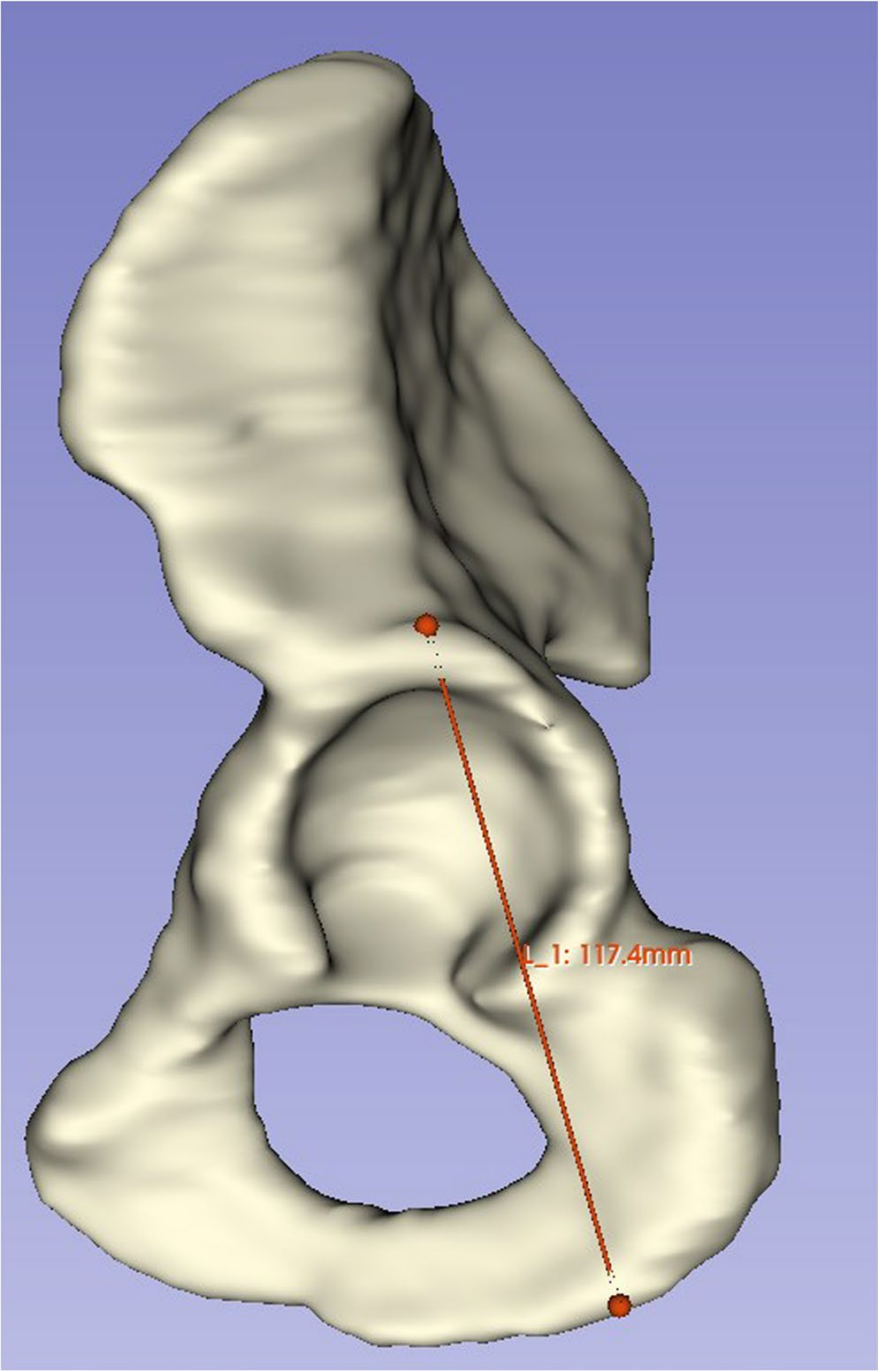
ISMM: Post-acetabular ischium length

**Fig. 6:**
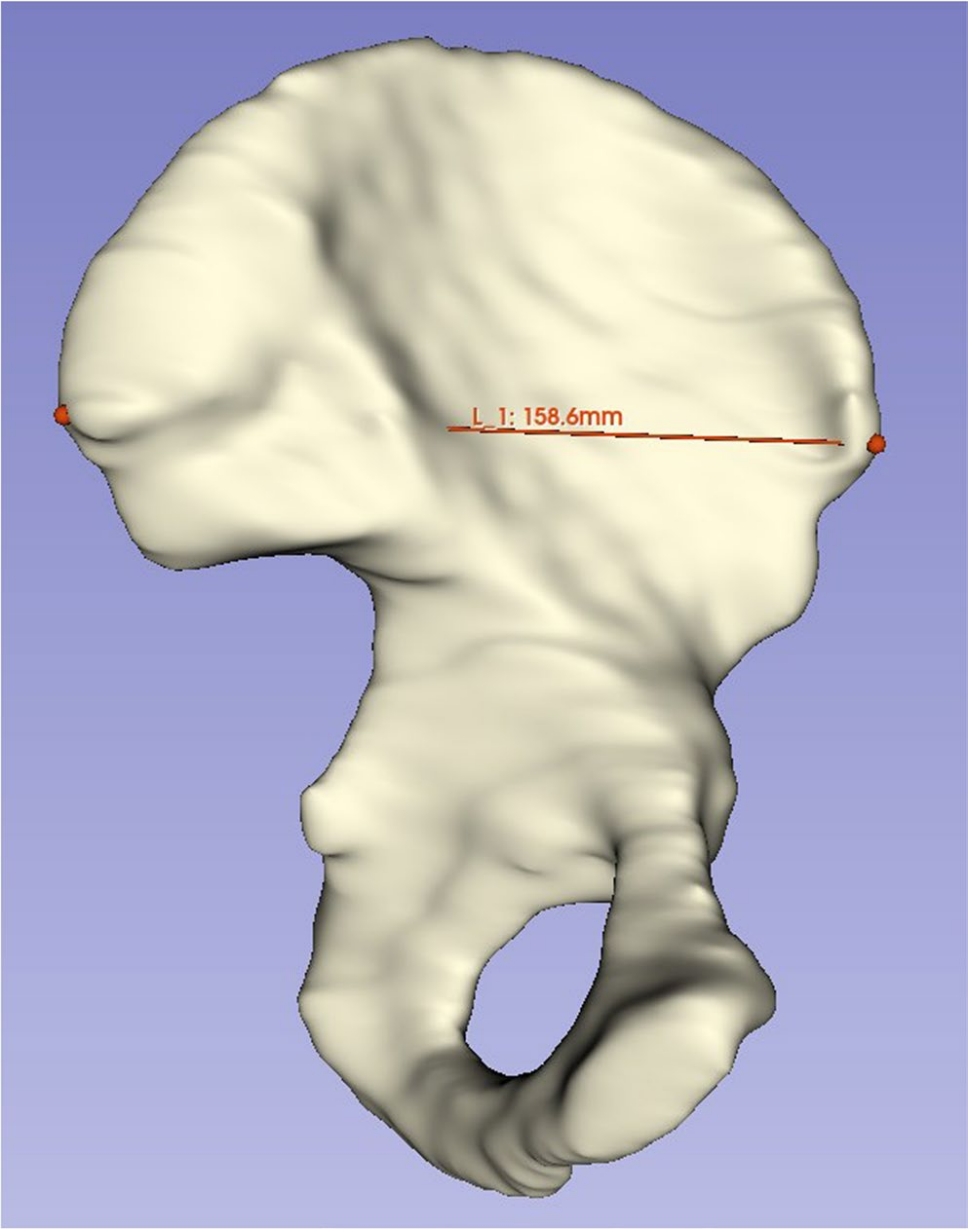
SCOX: Iliac breadth

**Fig. 7:**
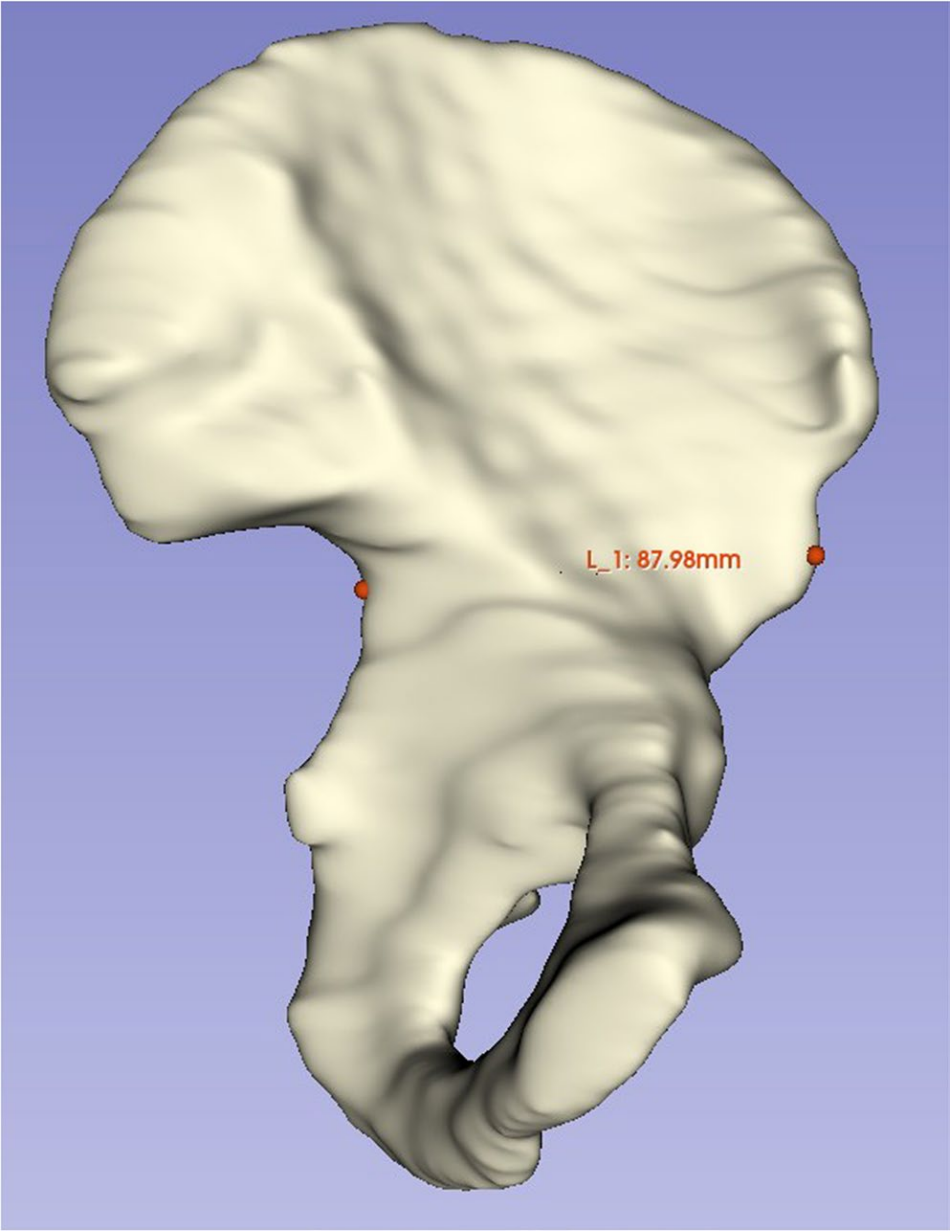
SS: Spino-sciatic length

**Fig. 8:**
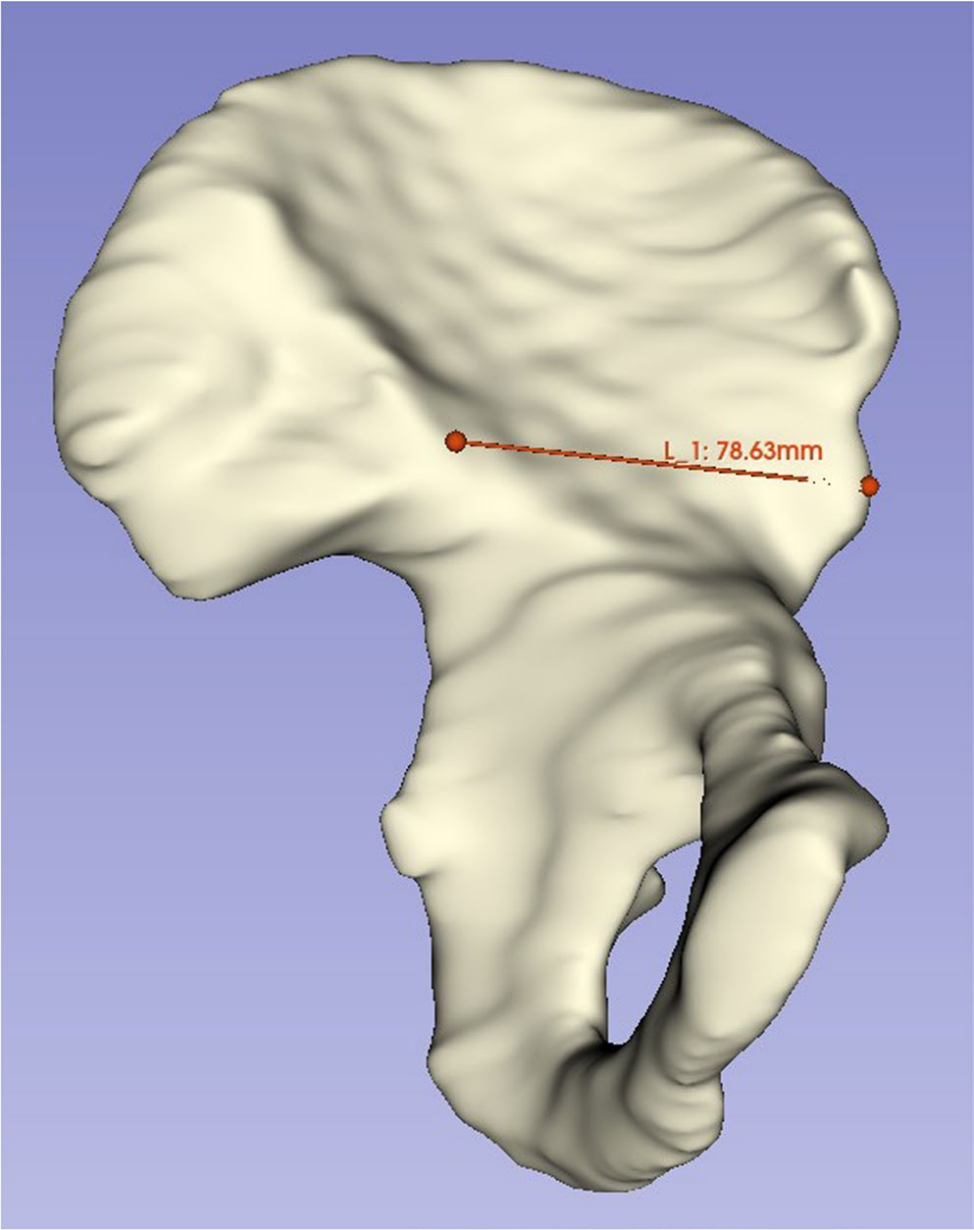
SA: Spino-auricular length

**Fig. 9:**
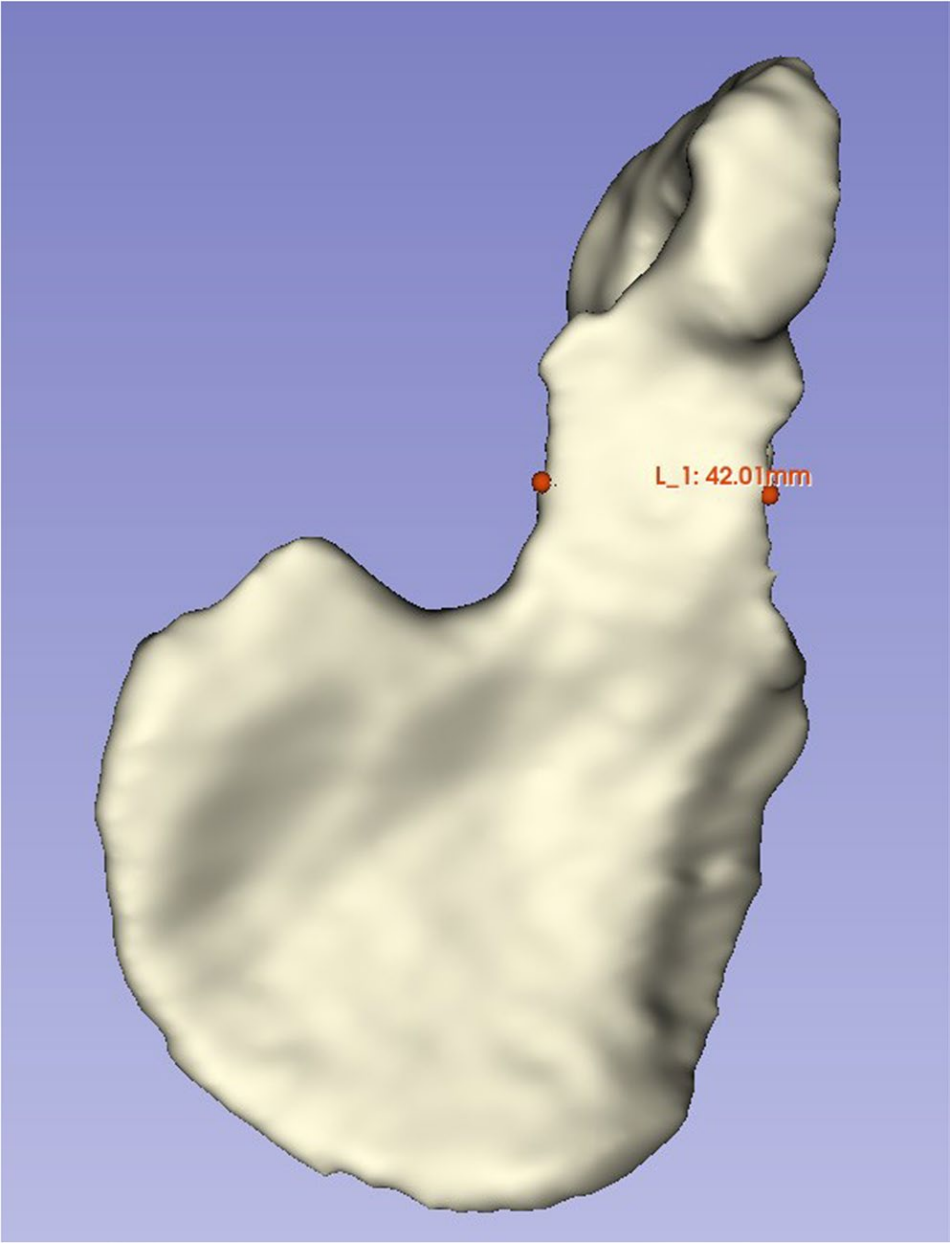
SIS: Cotylo-sciatic breadth

**Fig. 10:**
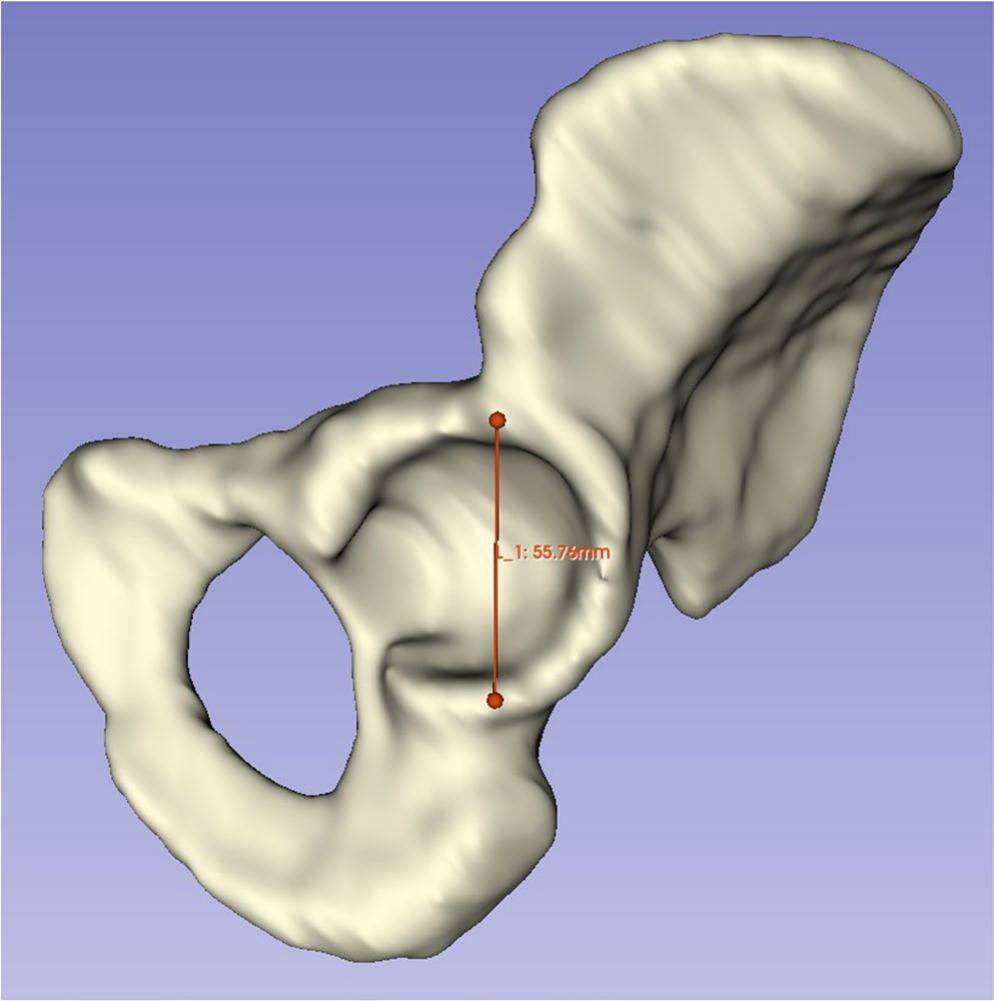
VEAC: Vertical acetabular diameter

In our study, we used computed tomography images from the archives of our Faculty’s Department of Radiology. Pelvic CT images of 100 men and 100 women aged 25–50, taken retrospectively between 2021 and 2025, were used. Slicer 5.8.0 software was used to create a three-dimensional representation of the bone and perform the measurements. Morphometric measurements were taken on the os coxae to estimate the sex of the individuals from the images (Figs. 1, 2, 3, 4, 5, 6, 7, 8, 9 and 10). Researchers have generally performed measurements on the left coxae in various populations, and Decker et al. reported no difference between the two sides [6, 7, 9, 10]. We performed measurements only on the left coxae to facilitate comparisons. This research was approved by the University Ethics Committee (Protocol no: 2025-SAEK-0173) and conducted in accordance with the Declaration of Helsinki. Using the obtained morphometric measurements, the sex of the bones was estimated using DSP2 software. The accuracy and validity of the DSP2 software were evaluated with the obtained data.

The study included 100 males, ages 25 to 47, with a mean age of 36.95 ± 6.75 years, and 100 females, ages 25 to 48, with a mean age of 38.66 ± 6.52 years. Images from patients with structural deformities that could affect the study results, previous surgical procedures, or a history of serious trauma due to traffic accidents were not included in the study. Because this was a population-specific study, foreign patients were not included. Measurements related to the 10 variables described in Table 1 were performed on the coxae, and the results were transferred to the DSP2 software. The software displayed probabilities for each bone and designated a bone as male or female when one of these probabilities was equal to or greater than 0.95. If the probabilities were less than 0.95, the sex was shown as “undetermined”.

**Table 1:** Definitions of 10 morphometric variables used in DSP2 (3)

PUM	Acetabulo-symphyseal pubic length	The minimum distance between the superomedial point of the pubic symphysis and the acetabular margin.	
SPU	Cotylo-pubic breadth	The pubic width, perpendicular to the main axis of the pubis between the most lateral point of the acetabulum and the medial aspect of the pubis.	
DCOX	Maximum pelvic height	The distance between the inferior margin of the os coxae and the most superior part of the iliac crest.	
IIMT	Height of the great sciatic notch	The distance between the posterior inferior iliac spine and the anterior margin of the greater sciatic notch.	
ISMM	Post-acetabular ischium length	The distance between the most anterior and inferior point of the ischial tuberosity and the most distal point on the acetabuli.	
SCOX	Iliac breadth	The distance between the anterior superior iliac spine and the posterior superior iliac spine.	
SS	Spino-sciatic length	The distance between the anterior inferior iliac spine and the deepest point of the greater sciatic notch.	
SA	Spino-auricular length	The distance between the anterior inferior iliac spine and the auricular point.	
SIS	Cotylo-sciatic breadth	The distance between the lateral border of the acetabulum and the midpoint of the anterior part of the greater sciatic notch.	
VEAC	Vertical acetabular diameter	The maximum vertical diameter of the acetabulum measured in accordance with the longitudinal axis of the ischium.	

Intraobserver reliability was analyzed (*n* = 20) by comparing the second and third sets of measurements (S2 and S3). Due to the use of a single, fixed rater, a two-way mixed-effects intraclass correlation coefficient (ICC) was calculated. The obtained ICC values were interpreted according to Koo and Li, where ICC values less than 0.5 indicate poor reliability, values between 0.5 and 0.75 indicate moderate reliability, values between 0.75 and 0.9 indicate good reliability, and values greater than 0.90 indicate excellent reliability [11]. Statistical analyses of the study were performed using the IBM SPSS Statistics 25.0 (IBM Corp., Armonk, New York, USA) statistical package program. Descriptive statistics of the variables (minimum value (min), maximum value (max), mean±standard deviation (mean ± SD) values) were calculated. The Shapiro-Wilk test was used to compare the data with a normal distribution. For comparison of data with normal distribution (dependent samples), the t-test was used, and for data that did not, the Mann-Whitney U test was used. The level of statistical significance was accepted as *p* < 0.05. The percentage of correct sex estimations and the percentage of incorrect results of the DSP2 program were calculated.

## Results

When the posterior probability threshold was set at 0.95 or higher, the sex of all individuals except three was estimated using DSP2’s 10 variables. The sexing success rate was 98.5%. The accuracy rate was 100%. When the posterior probability threshold was set at 0.90 or higher, the sexing rate of all individuals was estimated, resulting in a 100% sexing rate. There were no misidentifications, resulting in a 0% result. The accuracy rate was 100%.

In males, when the posterior probability threshold was set at 0.95 or above, the sexing rate was 100% when all DSP2 measurements (10 variables) were used. When the first eight variables were used, the sexing rate was 100%. When only the first four variables were used, the success rate was 98%. When only the last four variables were used, the success rate was 67%. The accuracy rate for all results obtained was 100%. Misdiagnosis, i.e., female identification, was 0%. When the posterior probability threshold was set at 0.90 or above, the sexing rate for the first four variables increased to 100%, and when the last four variables were used together, the sexing rate increased to 81%. When the posterior probability threshold was set at 0.85 or above, the sexing rate for the last four variables increased to 86%.

In women, when the posterior probability threshold was evaluated with a probability of 0.95 or higher, the sexing rate was found to be 97% when all 10 variables in DSP2 were used, 97% when the first 8 variables were used, 79% when the first 4 variables were used, and 36% when the last 4 variables were used. For the last 4 variables, sex estimation was incorrect in two individuals (2%). In other words, the accuracy rate for the last 4 variables was 94%. When the posterior probability threshold was set at 0.90 or higher, the sexing rate reached 100% when the first 10 variables were evaluated together, 98% for the first 8 variables, 85% for the first 4 variables, and 50% for the last 4 variables. When the posterior probability threshold was set at 0.85 or higher, the sexing rate increased to 100% for the first 8 variables, 91% for the first 4 variables, and 60% for the last 4 variables. Detailed information regarding sexing rate and accuracy rate for different posterior probability thresholds is provided in Table 2.

**Table 2:** Sexing rates and accuracy rates for DSP2 variables according to different posterior probability threshold values

	Sexing rate	Accuracy rate	Sexing rate	Accuracy rate
DSP2 variables	Male		Female	
	Posterior probability threshold 0.95			
All variables	100%	100%	97%	100%
First 8 variables	100%	100%	97%	100%
First 4 variables	98%	100%	79%	100%
Last 4 variables	67%	100%	36%	94%
	Posterior probability threshold 0.90			
All variables	100%	100%	100%	100%
First 8 variables	100%	100%	98%	100%
First 4 variables	100%	100%	85%	100%
Last 4 variables	81%	100%	50%	90%
	Posterior probability threshold 0.85			
All variables	100%	100%	100%	100%
First 8 variables	100%	100%	100%	100%
First 4 variables	100%	100%	91%	100%
Last 4 variables	86%	100%	60%	90%

Based on the values reported in the Methods section, excellent reliability was observed in the intraobserver analysis for all DSP variables (Table 3).

**Table 3:** Intraclass correlation coefficient (ICC) for intraobserver error analysis for the 10 variables of DSP methodology (*N* = 20)

Variable	ICC (Single)	95% IC (Single)	ICC (Average)	95% IC (Average)	*p*-value
PUM	0.955	0.891–0.982	0.977	0.942–0.991	< 0.001
SPU	0.934	0.841–0.973	0.966	0.914–0.986	< 0.001
DCOX	0.993	0.982–0.997	0.996	0.991–0.999	< 0.001
IIMT	0.961	0.904–0.984	0.980	0.949–0.992	< 0.001
ISMM	0.993	0.981–0.997	0.996	0.991–0.999	< 0.001
SCOX	0.991	0.976–0.996	0.995	0.988–0.998	< 0.001
SS	0.990	0.974–0.996	0.995	0.987–0.998	< 0.001
SA	0.918	0.805–0.967	0.957	0.892–0.983	< 0.001
SIS	0.942	0.859–0.976	0.970	0.924–0.988	< 0.001
VEAC	0.956	0.893–0.982	0.978	0.943–0.991	< 0.001

The minimum and maximum values ​​for each of the ten DSP variables obtained in the current study were measured within the range reported in the original study by Bruzek et al. (3). Descriptive statistics for sex-related dimorphism of DSP variables are shown in Table 4. In general, higher mean values ​​were found in males than in females, except for PUM and IIMT; the opposite was observed for PUM and IIMT. All variables, except SA, exhibited sex-related dimorphism (*p* < 0.001).

**Table 4:** Descriptive statistics (by sex) for the os coxae variables (*N* = 200, 100 male and 100 female)

Variable	Male (Mean ± SD)	Female (Mean ± SD)	t(df)	*p*-value	Cohen’s d
PUM	67.30 ± 3.92	70.74 ± 4.29	-5.91 (198)	< 0.001	-0.83
SPU	31.48 ± 2.44	25.70 ± 2.34	17.10 (198)	< 0.001	2.41
DCOX	223.21 ± 9.80	203.50 ± 9.32	14.57 (197)	< 0.001	2.06
IIMT	47.41 ± 5.63	55.25 ± 4.19	-11.17 (182)	< 0.001	-1.58
ISMM	115.79 ± 5.07	102.97 ± 5.12	17.81 (198)	< 0.001	2.52
SCOX	162.59 ± 8.22	158.42 ± 7.62	3.72 (198)	< 0.001	0.52
SS	80.15 ± 4.66	71.71 ± 4.14	13.53 (198)	< 0.001	1.91
SA	74.06 ± 5.97	74.12 ± 6.79	-0.07 (198)	0.947	-0.01
SIS	41.55 ± 2.94	36.26 ± 2.70	13.27 (198)	< 0.001	1.88
VEAC	55.67 ± 3.20	49.46 ± 2.69	14.84 (198)	< 0.001	2.10

*independent samples T test

## Discussion

This is the first study to examine the sex estimation rate and accuracy using DSP2 on pelvic CT images in a contemporary Turkish population. Using sets consisting of all 10 variables, the first 8 variables, and the first 4 variables of DSP2, very high sex estimation rates and accuracy were achieved for all three sets. Even when only the last 4 variables were used, although the sex estimation rate decreased, the decrease in accuracy rates remained minimal.

Bruzek and colleagues reported a sexing rate of 90.84% ​​and an accuracy rate of 99.65% when all 10 variables of DSP2 were used together. The sexing rates in our study were higher than their results. Bruzek and colleagues reported the sexing rate and accuracy for the last four variables in DSP2 as 40.44%-99.53% in men and 42.54%-98.52% in women, respectively [3]. In our study, the sexing rate and accuracy for the last four variables were 67%-100% in men and 36%-94% in women. These results, obtained from our country’s population, indicate that DSP2 provides better sex estimation results than their results.

In a study conducted on bones in Greece, Kranioti et al. reported a sexing rate of 88% and an accuracy rate of 97.43% when using a posterior threshold of 0.95 and 10 DSP variables. They also found an accuracy rate above 97% in all groups, based on the F1, F2, F3, and F4 groupings. While their sexing rate results are lower than ours, the results of our studies are similar in terms of accuracy [6].

In a study conducted by Marta San Millan and colleagues in Spain, using 10 variables of DSP2 for the left side, the sexing rate was 93.55% and the accuracy rate was 98.85 [4]. The results in our study were 98.5% and 100%, respectively, and were better than their results. When only male coxae were evaluated in our study, our results were 100% and 100%, respectively. In total, the failure to estimate sex in three of the 200 individuals in our study was in female coxae. However, Marta San Millan and colleagues reported that DSP2 was more successful in estimating sex in women than in men. This finding is the opposite of the results in our study. This may be due to several factors. One of these may be the different sample sizes in the two studies.

In our study, the sexing rate for the first four variables was found to be significantly lower in women than in men (79% vs. 98%). However, the accuracy rate was 100% for both genders. When the posterior probability threshold was lowered to 0.85, the sexing rate in women increased to 91%. Marta San Millan et al. found the sexing rate for the first four variables to be 95.8% in women and 83.5% in men [4]. While the sexing rate in their study was higher in women than in men, the situation in our study is quite different. The sexing rate in women is lower than in men. In terms of accuracy, their study achieved 100% for women and 95.65% for men, while our study found an accuracy rate of 100% for both genders. The findings of Mestekova et al.‘s study in France are partially similar to our findings [8]. They reported rates of 96.2% for men and 85.2% for women for the first four variables. The reason why the rates for women are lower in their study, as in ours, may be that the mean values for the IIMT variable in both studies are higher than in some other studies in the literature. They reported mean values for the IIMT variable as 44.80 ± 4.81 for men and 51.16 ± 5.94 for women. In our study, the mean values obtained for the IIMT variable were found to be slightly higher in both men and women. These higher values may explain why the results obtained for women are slightly lower. In addition, the mean values for the IIMT variable may be due to societal differences. In a study conducted by Ribennardo and Taylor on American whites, the mean values obtained for the IIMT variable were reported as 48.8 ± 4.6 for men, and these mean values are higher than the mean values for men in our study [12]. Similarly, in a study conducted by Knecht et al. on a French sample, the mean value reported for the IIMT variable in men was 49.96 ± 7.12 [13]. There may be many reasons for the differences in results between the literature for the IIMT variable. Congenital variations or age-related changes in the posterior inferior iliac spine used as a reference point, population-specific anatomical differences, geographical factors may be the reasons for differences in the measurement values of the IIMT variable.

Martin San Millan and colleagues, using the four variables of DSP2 that yielded the worst results, reported a sexing rate of 37.50% and an accuracy rate of 100% in women, and a sexing rate of 49.25% and an accuracy rate of 90.91% in men [4]. In our study, these values ​​were 36% and 94% in women and 67% and 100% in men, respectively. In our study, men were found to have better results in terms of accuracy, while in their study, women were found to have better results. The descriptive statistics in our study are similar to those in the Spanish population [4]. They also found higher mean values ​​in men than in women, except for PUM and IIMT. In left-sided coxae measurements, they report that the mean values ​​of PUM and SA did not show any sex-related dimorphism [4]. In our study, there was no difference between the sexes only in the SA value. The difference between the sexes was significant for the other nine variables (*p* < 0.05).

In a study conducted by Stan and colleagues in Romania on CT images, only four of the 10 variables used in DSP2 were selected. They measured PUM, SCOX, ISMM, and VEAC. They found a sexing rate of 85% in women, 57.50% in men, and 71.25% overall. The sexing rate was higher in women than in men. They found an accuracy rate of 100% in women and 95.65% in men, with a total of 98.24% [5]. It is not possible to fully compare our study with theirs. In our study, we investigated the first eight variables of DSP2, the first four, and the last four. Their lower sexing rate in their study is likely due to the variables they selected. Of the variables they selected, VEAC is one of the last four variables with the worst sexing rate in our study. In our study, the sexing rate decreased significantly when the last four variables were used. Furthermore, in our study, the accuracy rate decreased in women, dropping to *94*% when the last four variables were used.

In a study conducted in France on CT images of 106 individuals, Mestekova and colleagues reported a sexing rate of 92.3% in men and 97.2% in women using all DSP2 variables, with an accuracy rate of 100%. They achieved the lowest success rate in the group they selected for sexing rate, set 3 (IIMT, SS, SA, and SIS). Although set 3 was significantly lower in sexing rate (26.9% in males and 37% in females), the accuracy rate remained at 100% [8]. In our study, the worst results were obtained for the set using the last four variables. Although the sexing rate was slightly higher than in set 3, there was a misidentification of sex in women (2 person) in our study, with the accuracy rate dropping to 94%.

Paz and colleagues tested DSP2 in 116 CT scans in the Danish population. They reported sexing rates for the 10 variables, the first eight variables, the first four variables, and the last four variables in DSP2 as 93.9%, 93.1%, 81.9%, and 49.7%, respectively [9]. In our study, the same rates were 100%, 100%, 98%, and 67% in men and 97%, 97%, 79%, and 36% in women. The sexing rate for DSP2 in our population is higher. The success rate for the last four variables is also higher. Based on these results, it could be argued that the DSP2 results are more successful in our population. However, the differences in sample sizes between studies could have influenced the results.

## Conclusion

The DSP2 method can be reliably used for sex estimation in the modern Turkish population through measurements taken from CT images. For both the 10 variables and the first 8 variables of DSP2, the sexing rate was found to be 98.5% and the accuracy rate was 100%. Although the sexing rate decreased slightly in women for the first 4 variables, the accuracy rate was 100% for both men and women. Studies using DSP2 on CT images are limited in the literature. The success of DSP2 in sex estimation should be evaluated through measurements performed on CT images from populations living in countries on different continents and in different geographical regions.
